# Development and Validation of Non simultaneous Retinal Image Acquisition–Based Retinal Oximeter

**DOI:** 10.1038/s41598-017-04085-x

**Published:** 2017-06-27

**Authors:** Sumeer Singh, Gunasekaran Velu, Rajiv Raman

**Affiliations:** 1 Elite School of Optometry, Saint Thomas Mount, Chennai, Tamil Nadu India; 2 Birla Institute of Technology and Science, Pilani, India; 3 Cynaptix Technologies Private Limited, Chennai, Tamil Nadu India; 4 Shri Bhagawan Mahavir vitreo Retinal Services, Sankara Nethralaya, Chennai, Tamil Nadu India

## Abstract

The purpose of this study was to develop and validate a tool for the measurement of retinal oxygen saturation using a conventional fundus camera–based nonsimultaneous imaging technique. Retinal oximetry setup comprising a conventional Zeiss FF450IR fundus camera, dual wavelength band-pass filters of wavelengths 570 and 600 nm were used. Image analysis was performed using MATLAB R2013b. All the study participants underwent comprehensive eye examination, fundus examination, complete hemogram analysis, and evaluation of systemic hemodynamics. Fundus images were captured by a nonsimultaneous retinal oximetry. A total of 45 subjects were included in the analysis. Median age of the subjects was 21 years ranging from 19 to 34 years. The median retinal arteriolar and venular oxygen saturation was 94.7% and 55.8%. Comparison of retinal oxygen saturation between three visits showed no statistically significant difference for both arteriolar (p = 0.33)and venular oxygen saturation (p = 0.79). Intraclass correlation coefficients for test–retest, short-term, and day-to-day repeatability were 0.84, 0.90, and 0.86 for arteriolar oxygen saturation and 0.92, 0.98, and 0.98 for retinal venular oxygen saturation. Oxygen saturation in retinal arteriolar and venular blood vessels can be measured by nonsimultaneous image acquisition technique using a conventional fundus camera with good repeatability.

## Introduction

It is common that retinal vascular diseases affect retinal hemodynamics, which in turn alter the delivery of oxygen to the ocular tissues and cause severe visual impairment. The need for early detection of retinal hypoxia in the early stages of the disease has led to the development of retinal oximetry for measuring the oxygen saturation in retinal arteries and veins^1^. It has been used in studying the pathophysiology of various ocular conditions such as diabetic retinopathy, glaucoma, retinitis pigmentosa, and retinal ischemic conditions^2–5^. Oxymap retinal oximeter (Oxymap ehf., Reykjavik, Iceland) and retinal vessel analyzer (RVA, Imedos Systems UG Jena, Germany) are two commercially available retinal oximeters^1, 6^. Mohan *et al*.^7^ used Oxymap retinal oximetry to describe the normative database for retinal oximetry in Asian Indian eyes (mean arteriolar oxygen saturation was 90.3% ± 6.6% and the venular oxygen saturation was 56.9% ± 6.3%).

Although in recent years, the use of these oximeters in research has increased, they are not utilized in clinical practice. This is probably because of the fact that these measurements are more for academic interest and the equipment are stand-alone devices used only for this purpose and need change in the hardware part of the imaging system. These are usually camera specific and as they are based on the simultaneous acquisition of retinal image with two wavelength filters, they need changes in the camera, which increases the cost; thus, again limiting their clinical use.

The aim of this study was to develop a method for measuring oxygen saturation in the retinal vessels using the existing retinal camera, Zeiss FF450IR (Carl Zeiss Meditech, Germany); to elucidate the test–retest, day-to-day, and short-term variability of the system; and to report the results of the pilot study in using this customized device.

## Materials and Methods

### Development of retinal oximeter

**Figure 1:**
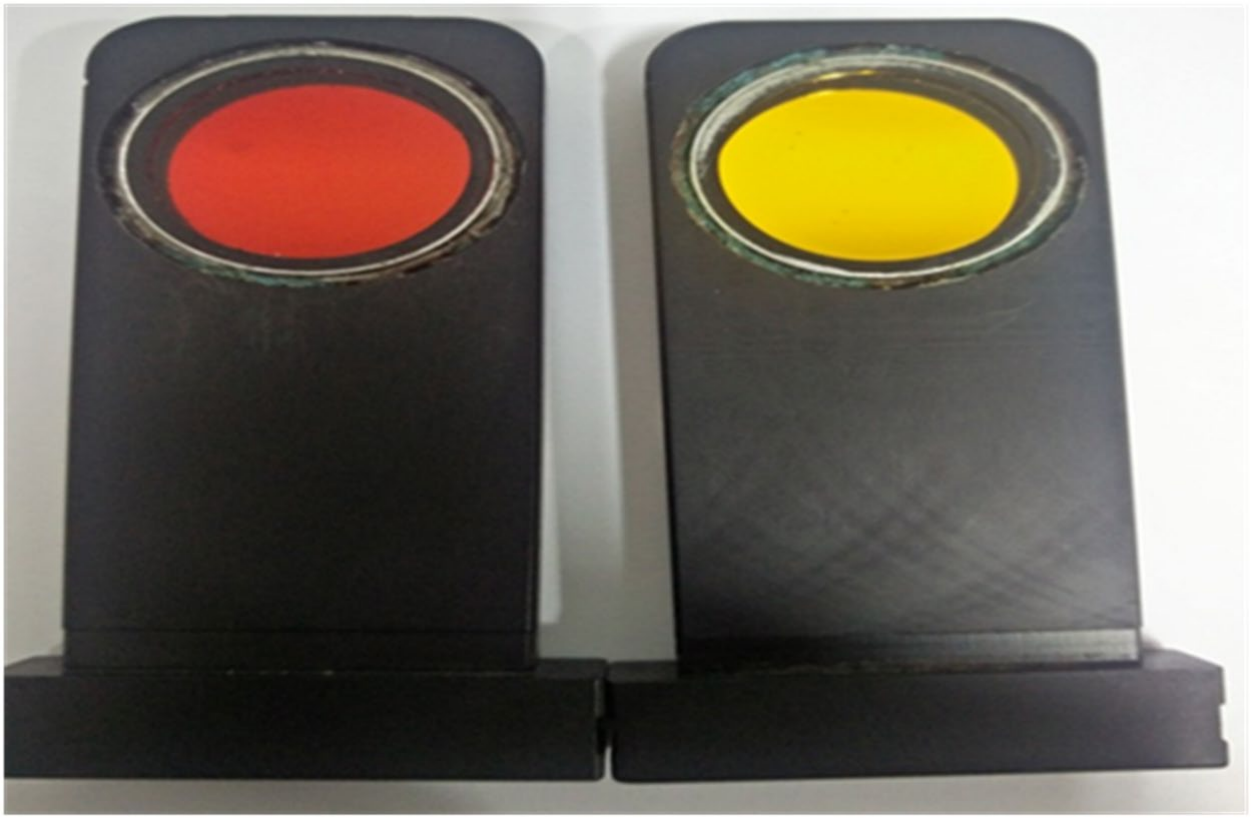
Manual Filters of wavelength 570 and 600 nm used for capturing the retinal images.

In this study, development of retinal oximeter was based on the nonsimultaneous image acquiring technique. Zeiss FF450IR (Carl Zeiss Meditech) fundus camera with Zeiss ZK-5 camera back was used for capturing the retinal images using oxyhemoglobin sensitive and oxyhemoglobin insensitive wavelengths, the wavelengths of filters that were used for the oxyhemoglobin sensitive and oxyhemoglobin insensitive wavelengths were 600 and 570 nm with a bandwidth of 10 nm each^8^ (Fig. 1); The individual has given written informed consent to publish his photograph in the manuscript.

**Figure 2:**
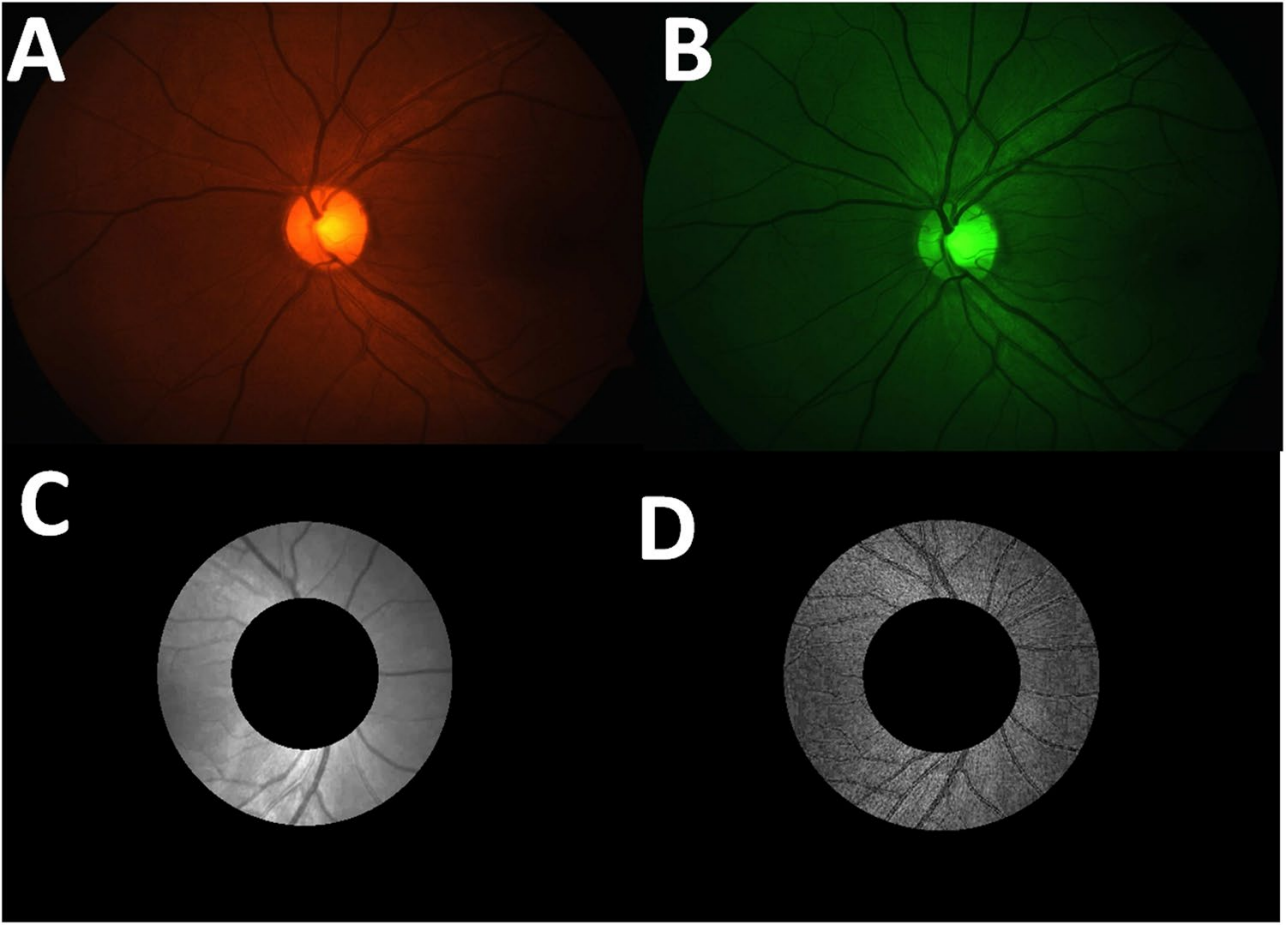
Image Processing Regions from the Captured Fundus Images. (**A**) Image captured with oxyhemoglobin sensitive wavelength. (**B**) Image captured with oxyhemoglobin insensitive wavelength. (**C**) Image processing region of the oxyhemoglobin sensitive wavelength image for the measurement of optical density. (**D**) Image processing region of the registered oxyhemoglobin insensitive wavelength image for the measurement of optical density.

An algorithm was written using MATLAB R2013b to calculate the optical density ratio of the blood vessels (optical density is the ratio of the brightness inside and outside the vessels) at two different wavelengths, as it is linearly proportional to the oxygen saturation. Image registration was done between the images captured with oxyhemoglobin sensitive and insensitive wavelength filters to compensate for the micro-saccadic eye movements caused during the imaging using the intensity-based automatic image registration in MATLAB. The captured image was converted from RGB to gray scale and the morphological functions such as dilation and erosion were applied for the preprocessing of the image and then threshold analysis was done to identify the optic disc contour. A concentric inner circle of one disc diameter, which was optic disc-centered circle and a concentric outer circle, which was two times the diameter of the optic disc-centered circle were created in the fundus images. The region between the inner and outer concentric circles defined the measurement region in which the vessels were processed(Fig. 2). Contour width and contour area were defined to identify the vessels; the larger contour width was identified as vein and the smaller width as artery. Optical density was calculated as the logarithmic ratio of fundus reflectance inside and just outside the vessels. Depending on the vessel width, the reflectance value was taken inside the vessel wall at 75–100 locations automatically along with a similar number for the outside vessel wall points. The average of these was taken at each of the vessels to calculate the optical density. The calibration technique used in this study was based on the work carried out by Hardarson *et al*.^1^.

### Imaging technique

All the subjects’ eyes were dilated using a combination of 5% phenylephrine and 0.8% tropicamide drops and the images were captured in a dark room, with the only external light source being the computer screen. Throughout the study period, all the images were captured by a single experienced photographer. Both the eyes of the subjects were imaged with the image of the right eye taken first, followed by the left eye. All the images were captured with a disc-centered 50 degree field of view. The filters were manually changed by the investigator in the external slot of the Zeiss FF450IR fundus camera. Oxyhemoglobin sensitive wavelength image was captured first followed by the oxyhemoglobin insensitive wavelength image. The time gap between each flash or image was 1 minute. Ten images were captured for each eye (five using oxyhemoglobin sensitive wavelength and five using oxyhemoglobin insensitive wavelength). Images with poor focus, poor contrast, or poor positioning were excluded and recaptured.

### Pilot study

#### Study population

The study design was a prospective cross-sectional study conducted between August 2015 and April 2016. The institutional review board and the ethics committee of Vision Research Foundation, Chennai, India, approved the study and the study followed the tenets of Declaration of Helsinki (Letter attached). A total of 50 healthy subjects were enrolled in the study after a written informed consent was obtained from all the subjects before enrolling them into the project. All participants were healthy and nonsmokers, without any history of systemic illness or medications, which can affect the blood flow. All the subjects had no previous history of ocular disease or surgery; they had a refractive error less than ± 6.00D and a cylindrical error less than 3.00D.

#### Study protocol

All subjects underwent detailed comprehensive eye examination that included detailed history taking, visual acuity examination with internally illuminated Snellen chart, objective refraction using a streak retinoscopy followed by a subjective refraction, extraocular motility, and cover test. Slit lamp biomicroscope examination and intraocular pressure measurement were carried out using Goldmann applanation tonometry. The subject’s eyes were dilated using a combination of 5% phenylephrine and 0.8% tropicamide drops. All the subjects underwent dilated fundus examination with indirect ophthalmoscopy by a retina specialist.

For the complete hemogram analysis, 3 mL of venous blood was collected into vaccutainer tubes containing K2 EDTA (ethylene diamine tetra acetic acid)and ESR (erythrocyte sedimentation rate) tubes containing 3.8% sodium citrate by a non traumatic venepuncture. EDTA samples were run in the LH750 Beckman Coulter’s fully automated hematology analyzer and ESR tubes were run in Ves-Matic20 (fully automated) ESR analyzer and were measured using an automated sedimentation technique. Complete hemogram analysis included the measurement of hemoglobin, packed cell volume, total red blood cell count, mean corpuscular hemoglobin, mean corpuscular hemoglobin concentration, percentage of basophils, eosinophils, lymphocytes, monocytes, neutrophils, total count, and ESR measurement. Blood pressure (BP) and heart rate were recorded using a digital automatic BP monitor (Omron HBP-1300; Omron, Netherlands, Europe) for all the subjects after 15 minutes of resting period. This was done to eliminate the exercise-induced fluctuations in the readings. An average of three readings was taken as the mean BP of an individual. Peripheral arteriolar oxygen saturation was measured using a finger pulse oximetric device. The sensor was attached to the index finger of the subjects and the output was displayed on the screen.

### Repeatability testing

#### Test–retest repeatability

Test–retest repeatability was calculated for the five sets of images captured during the baseline visit.

#### Short-term repeatability

After the baseline measurements, a resting period of 20–25 minutes was given and five sets of images were captured to assess the short-term repeatability.

#### Day-to-day repeatability

After 1 week, an identical study was performed to assess the day-to-day repeatability on the same subjects.

## Results

In the pilot study of the 50 subjects, five subjects(10%)were excluded from the study as the software was unable to detect the vessel wall contour because of poor image quality. Table 1 summarizes the demographic, ocular, systemic, and hematological parameters of the study population. The subjects were young (average age: 21 years), emmetrope, normotensive (average BP: 116/68 mm Hg). They had no ocular abnormalities. The average oxygen saturation as measured by finger oximetry was 97% (interquartile range: 97–98%). They had no hematological abnormalities.

**Table 1:** Demographic, Ocular, and Systemic Parameters of the Study Population.

Variables	Median (IQR), n = 45
Age, years	21 (20, 23)
Gender (Male: Female)	20:25
Refractive error (Diopters)	0 (−0.87, 0)
Intraocular pressure, mm Hg	14 (12, 14)
Systolicblood pressure, mm Hg	116 (110, 120)
Diastolic blood pressure, mm Hg	68 (63, 73)
Pulse/minute	75 (70, 83)
Oxygen saturation (%)	97(97, 98)
Ocular perfusion pressure, mm Hg	42.78 (39.11, 45.67)
Hemoglobin (g/dL)	13.1 (12.4, 14.9)
Packed cell volume (%)	40.5 (37.7, 45.1)
Total red blood count (10^3)	4.71 (4.39, 5.2)
Mean corpuscular hemoglobin (picograms)	28.2 (27.5, 29.9)
Mean corpuscular hemoglobin concentration (%)	32.5 (32.2, 32.8)
Basophils (%)	0 (0, 0)
Eosinophils (%)	3 (2, 5)
Lymphocytes (%)	35 (30, 38)
Monocytes(%)	6 (4, 7)
Neutrophils (%)	55 (53, 60)
Total count (10^3)	8.3 (7.8, 9.2)
Erythrocyte sedimentation rate (mm)	11 (7, 21)
Reticulocytes (%)	0.8 (0.7, 0.9)

IQR,interquartile range; mm Hg, millimeter of mercury.

To understand the laterality between the eyes, Wilcoxon signed rank test was used. No statistical significant bilateral differences were noted for both the retinal arteriolar (p = 0.80) and venular oxygen saturation (p = 0.34). Thus, only the eyes in the right side were considered for further statistical analysis.

The median retinal oxygen saturation of the study sample (n = 45) was 94.7% in retinal arterioles and 55.8% in the retinal venules. The median arteriovenous difference was found to be 39.8%. No statistical significant difference was found between the genders for retinal arteriolar and venular oxygen saturation (p = 0.19 and 0.94, respectively, Mann–Whitney U test). Retinal oxygen saturation measurements were compared between three visits. Friedman test was performed to observe the differences in retinal oxygen saturation values between the visits, and no statistically significant difference was found (Table 2). No association was seen between the retinal oxygen saturation and any of the ocular, systemic, and hematological parameters.

**Table 2:** Median Retinal Oxygen Saturation Values between the Visits.

Retinal oxygen saturation	Baseline	After 20 minutes	After 1 week	p-Value
	Median (IQR)	Median (IQR)	Median (IQR)	
Arteriolar oxygen saturation (%)	94.7 (94.0, 95.3)	94.5 (93.3, 95.2)	94.8 (94.1, 95.4)	0.33
Venular oxygen saturation(%)	55.8 (52.1, 58.9)	56.6 (52.4, 60.1)	56.5 (52.2, 58.7)	0.79
Arteriovenous difference (%)	39.8 (35.3, 44.2)	37.6 (35.0, 42.9)	38.5 (35.4, 42.4)	0.65

IQR, interquartile range.

Intraclass correlation coefficient(ICC) was calculated for the test–retest, short-term, and day-to-day repeatability measurements, and it showed a good correlation between the visits (Table 3).

**Table 3:** Intraclass Correlation Coefficients for the Repeatability Measurements in Healthy Subjects.

	Test–retest repeatability	Short-term repeatability	Day-to-day repeatability
ICC (retinal arteries)	0.84	0.90	0.86
ICC (retinal veins)	0.92	0.98	0.98

ICC, intraclass correlation coefficient.

## Discussion

In the last few years, several techniques that assisted in studying retinal oximetry in animal and human eyes have evolved^1, 9^. In this study, we developed a dual wavelength retinal oximetry and evaluated the repeatability of this fundus camera–based nonsimultaneous image acquisition technique that may assist in the future clinical investigations of retinal oximetry in various retinal pathologies.

Kim *et al*.^10^ used dual wavelengths of 568 and 600 nm for the oxygen saturation measurements, similar to this study. Although there were no differences in the retinal arteriolar and venular saturation levels between the studies, the prototype developed by Kim *et al*. used a measurement region closer to the disc (0.6 and 1 disc diameter); the reflections from the optic disc might falsely influence the retinal oxygen saturation levels. This has been taken care of in this study by choosing the measurement region between one and two disc diameters.

Geirsdottir *et al*.^11^ reported a mean arterial oxygen saturation of 90.4% ± 4.3% in healthy individuals aged 18–80 years, the arteriolar oxygen saturation values were 4% lower than that reported in this study (94.7%) of healthy individuals aged 18–35 years. This difference could probably be attributed to the presence of an older age group in the earlier study. Our results were in agreement with the study conducted on multiethnic background subjects by Jani *et al*.^8^. Mean arterial oxygen saturation of arteries (90.4% ± 4.3%) was 4% lower when compared with our results, and mean venular oxygen saturation(55.3% ± 7.1%) was similar to ours. Ethnicity driven differences were reported by Jani *et al*.^8^ who found an increase in the retinal arteriolar oxygen saturation in Asian eyes as compared with the eyes of the subjects from African–American, Caucasian, and Hispanic ethnicities.

In India, Mohan *et al*.^7^ used Oxymap retinal oximetry and had described normative database for retinal oximetry in Asian Indian eyes. Subjects of the age group between 18 and 63 years were studied. The mean arteriolar oxygen saturation was 90.3% ± 6.6% and the venular oxygen saturation was 56.9% ± 6.3%, which was similar to the values reported in this study. The arteriovenous differences found in their study matched with this study (39.8%).

The oxygen saturation values reported in our study were also in agreement with the normal oxygen saturation values reported in the literature using Imedos simultaneous imaging system, which accounts for the fundus pigmentation. Man *et al*.^12^ reported 95.94% (range: 91.53–98.49%) arterial oxygen saturation and 62.35% (range: 57.65–64.17%) median venular oxygen saturation, which was 7% higher than that reported in this study. Table 4 summarizes the retinal oxygen saturation levels in comparison with the existing literature^6, 8, 10, 11, 13, 14^.

**Table 4:** Retinal Oxygen Saturation Values in Healthy Individuals across the Literature.

Authors	Sample size	Retinal arteriolar oxygen saturation (%)	Retinal venular oxygen saturation (%)	Age of the subjects (years)	Oximetry
		Mean ± SD	Mean ± SD		
Current study, 2016	45	94.4, IQR	55.0, IQR	21 (18–35)*	Nonsimultaneous retinal oximetry
Kim *et al*.^10^	11	90.3 ± 6.6	55.3 ± 7.1	Not mentioned	Nonsimultaneous retinal oximetry
Geirsdottir *et al*.^11^	120	92.2 ± 3.7	55.6 ± 6.3	47 (18–80)*	Oxymap simultaneous oximetry
Lasta *et al*.^6^	20	91.0 ± 9	48.0 ± 11	23 ± 4	Imedos simultaneous oximetry
Jani *et al*.^8^	61	90.4 ± 4.3	55.3 ± 7.1	44.1 ± 14.7	Oxymap simultaneous oximetry
Yip *et al*.^13^	118	93.6 ± 6.9	54.2 ± 6.9	54.6 ± 6.86	Oxymap simultaneous oximetry
Kashani *et al*.^14^	45	93.0 ± 7	65.0 ± 5	61 ± 9	HCTIS simultaneous oximetry

HCTIS, hyperspectral computed tomographic imaging spectroscopy; IQR, interquartile range; SD, standard deviation. *Values in median (IQR).

In this study, no association was found between the retinal arteriolar and venular oxygen saturation with any of the systemic parameters. This finding was supported by the study undertaken by Yip *et al*.^13^, who found no association between the retinal arteriolar oximetry measurements and any of the systemic factors. Yip *et al*.^13^ also performed univariable analyses and found older age to be significantly associated with reduced venular oximetry levels.

This study also compared complete hemogram parameters with the retinal oxygen saturation levels. No correlation was seen between the retinal oximetry measurements and any of the complete hemogram parameters. Though oxygen saturation measurements were dependent on hemoglobin levels, no association was seen between them. The probable reason would be due to the homogenous group of healthy individuals with normal hemogram parameters recruited in this study and also we did not have a disease group with abnormal hemoglobin count to actually observe the association of complete hemogram with retinal oxygen saturation.

We also reported the test–retest, short-term, and day-to-day repeatability of the nonsimultaneous retinal oximetry. Yip *et al*.^13^ measured the intravisit repeatability of the Oxymap retinal oximeter from the two images taken on the same visit. The test–retest repeatability was 0.91 for arteries and 0.90 for veins, which is similar to our results. Lasta *et al*.^6^ measured the test–retest, short-term, and day-to-day repeatability of the Imedos retinal oximetry system. The ICC comparison of this study matched with the study conducted by Lasta *et al*.^6^.

The strength of the study is that we were able to reproduce earlier oximetry readings using other systems and achieved good reproducibility values near the optic disc. However there are certain limitations, firstly the need for two filters which need to be changed during measurements, secondly it needs a experienced photographer to take two sequential images of the same quality and illumination uniformly, third it needs a offline image registration and last it failed in 10% of measurements. The device was tested in normal subjects as a pilot study, its performance in retinal pathology like diabetic retinopathy, retinal ischemic conditions needs to be studied. Though this prototype seems to be consistent, further modification to address the above limitations would make it a more efficient device.

## Conclusion

We described the development and validation of a method for measuring oxygen saturation in retinal vessels using the existing retinal camera, Zeiss FF450IR (Carl Zeiss Meditech, Germany), which showed a good test–retest, day-to-day, and short-term variability and results of the pilot study showing oxygen saturation values consistent with other commercially available products.
